# Overexpression of the Mitochondrial T3 Receptor p43 Induces a Shift in Skeletal Muscle Fiber Types

**DOI:** 10.1371/journal.pone.0002501

**Published:** 2008-06-25

**Authors:** François Casas, Laurence Pessemesse, Stéphanie Grandemange, Pascal Seyer, Naïg Gueguen, Olivier Baris, Laurence Lepourry, Gérard Cabello, Chantal Wrutniak-Cabello

**Affiliations:** 1 INRA, UMR866 Différenciation cellulaire et croissance, Montpellier, France; 2 Université Montpellier 1, Montpellier, France; 3 Université Montpellier 2, Montpellier, France; Université de Lyon, CNRS, INRA, Ecole Normale Supérieure de Lyon, France

## Abstract

In previous studies, we have characterized a new hormonal pathway involving a mitochondrial T3 receptor (p43) acting as a mitochondrial transcription factor and consequently stimulating mitochondrial activity and mitochondrial biogenesis. We have established the involvement of this T3 pathway in the regulation of *in vitro* myoblast differentiation.We have generated mice overexpressing p43 under control of the human α-skeletal actin promoter. In agreement with the previous characterization of this promoter, northern-blot and western-blot experiments confirmed that after birth p43 was specifically overexpressed in skeletal muscle. As expected from *in vitro* studies, in 2-month old mice, p43 overexpression increased mitochondrial genes expression and mitochondrial biogenesis as attested by the increase of mitochondrial mass and mt-DNA copy number. In addition, transgenic mice had a body temperature 0.8°C higher than control ones and displayed lower plasma triiodothyronine levels. Skeletal muscles of transgenic mice were redder than wild-type animals suggesting an increased oxidative metabolism. In line with this observation, in gastrocnemius, we recorded a strong increase in cytochrome oxidase activity and in mitochondrial respiration. Moreover, we observed that p43 drives the formation of oxidative fibers: in soleus muscle, where MyHC IIa fibers were partly replaced by type I fibers; in gastrocnemius muscle, we found an increase in MyHC IIa and IIx expression associated with a reduction in the number of glycolytic fibers type IIb. In addition, we found that PGC-1α and PPARδ, two major regulators of muscle phenotype were up regulated in p43 transgenic mice suggesting that these proteins could be downstream targets of mitochondrial activity. These data indicate that the direct mitochondrial T3 pathway is deeply involved in the acquisition of contractile and metabolic features of muscle fibers in particular by regulating PGC-1α and PPARδ.

## Introduction

Skeletal muscle of vertebrates contain myofibers differing in contractile function, mitochondrial content and metabolic properties. Slow-twitch fibers are characterized by type I myosin heavy chain (MHC) expression and a high mitochondrial density leading to a prominent oxidative metabolism. Fast-twitch fibers express type II MHCs including three subtypes: IIa, IIx and IIb. Type IIb fibers display a reduced mitochondrial density associated with a principally glycolytic metabolism. The oxidative capacity of type IIa and IIx fibers are intermediate between that recorded in fibers Type I and IIb [1], [2].

In addition to its metabolic activity, triiodothyronine (T3) affects developmental processes, and is in particular considered as a major regulator of *in vivo* muscle development. This hormone not only stimulates growth of this tissue by increasing the number and diameter of muscle fibers [3], [4], but also regulates the transition between neonatal and adult myosin isoforms [5] and influences the contractile features of adult muscle fibers [6]. Thyroid hormone acts through nuclear receptors (T3Rs) encoded by the TRα and TRβ genes (NR1A1 and NR1A2 according to nuclear hormone receptor nomenclature) [7], [8]. These receptors are ligand-dependent transcription factors that constituvely bind to specific sequences called thyroid hormone response elements (T3RE) located in the promoter of T3 target genes.

More recently, we have identified in mitochondria two N-terminally truncated forms of the nuclear receptor TRα1, with molecular weight of 43 and 28 kDa (p43 and p28) [9], [10]. These proteins are synthesized by the use of internal initiation sites of translation occuring in the TRα1 transcript. Despite the occurence of a nuclear localization signal, p43 is specifically imported into the mitochondria according to an atypical process [11]. In gel shift experiments, p43 binds as dimeric complexes involving at least two other truncated forms of nuclear receptors located in mitochondria, mt-RXR and mt-PPAR, to specific sequences of the mitochondrial genome, sharing strong homologies with nuclear T3RE [11], [12], [13]. Consequently, on isolated mitochondria, p43 stimulates mitochondrial transcription and protein synthesis in the presence of T3 [11]. Lastly, in CV1 cells, p43 overexpression stimulates mitochondrial biogenesis and respiratory chain activity [9].

We have previously shown that mitochondrial activity is an important regulator of myoblast differentiation. While inhibition of mitochondrial protein synthesis by chloramphenicol impaired myoblast differentiation, stimulation of mitochondrial activity by p43 overexpression induced a potent stimulation of terminal differentiation [14], [15]. This regulation which does not involve changes in ATP stores, allows the expression of nuclear genes involved in the regulation of cell proliferation and differentiation. In particular, in myoblasts, p43 overexpression stimulates terminal differentiation, by down-regulating c-Myc expression and up regulating myogenin expression [14], [15]. In addition, it also induces a preferential expression of the slow myosin isoform, through increasing calcineurin levels [16].

These data led us to examine a potential role of the direct mitochondrial pathway in *in vivo* muscle development and phenotype. To assess the influence of this mitochondrial receptor, we have generated transgenic mice overexpressing p43 under the control of the human α-skeletal actin promoter (HSA), a skeletal muscle specific promoter 17, 18. Here we report, that this overexpression increases mitochondrial gene expression and mitochondrial biogenesis. In addition, p43 induces a shift toward the oxidative phenotype: in soleus, an oxidative muscle, MyHC IIa fibers were partly replaced by type I fibers; in quadriceps, an oxido-glycolytic muscle, we found an increase in the frequency of MyHC IIa and IIx fibers associated with a reduction in the number of glycolytic IIb fibers. These data indicate that the direct mitochondrial pathway is deeply involved in the acquisition of contractile and metabolic features of muscle fibers.

## Results

### Production of p43 overexpressing mice

**Figure 1:**
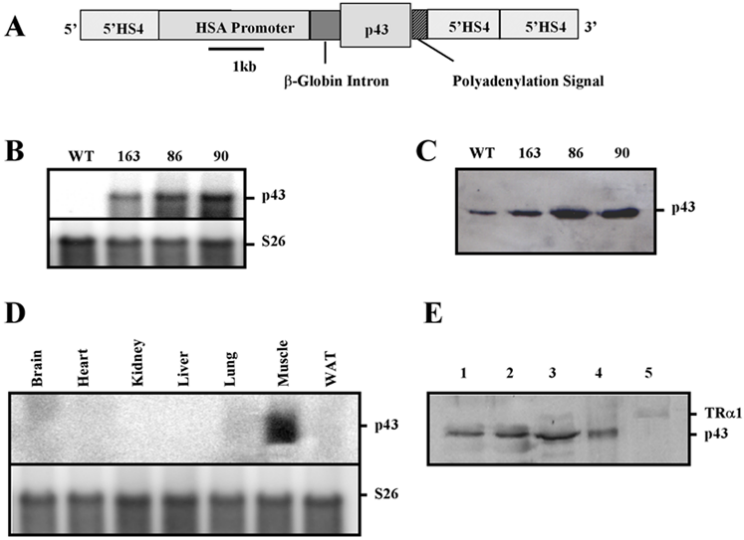
Transgenic expression of p43 in mouse skeletal muscle. (A) Schematic representation of the construct used for microinjection of [C57BL/6 x CBA] F1 fertilized oocytes. HSA: human α-skeletal actin; 5′HS4: chicken β-globin insulator. (B) mRNAs were isolated from quadriceps from transgenic mice of the 86, 90 and 163 lines versus wild-type (WT) animals and subjected to hybridization analysis with probes for p43. Hybridization with ribosomal S26 probes served as loading control. 20 µg of total RNAs were analyzed. (C) p43 protein levels in quadriceps muscle mitochondria from transgenic mice of the 86, 90 and 163 lines versus wild-type animals, visualized by western-blot using an antibody raised against TRα. 50 µg of mitochondrial proteins were analyzed. (D) mRNAs were isolated from various tissues from transgenic mice of the 86, 90 and 163 lines versus wild-type animals and subjected to hybridization analysis with probes for p43. Hybridization with ribosomal S26 probes served as loading control. 20 µg of total RNAs were analyzed. (E) Western blot of the fractions collected during the nuclear isolation using an antibody raised against TRα. Fractions are whole muscle homogenate-I (1), whole muscle homogenate-II (2), crude nuclear fraction (3), plasma membranes, mitochondria (4), nuclei (5). 25 µg of proteins were analyzed. Arrows indicate the nuclear receptor TRα1 and p43.

In order to assess the importance of p43 in the control of muscle fiber plasticity and of mitochondrial biogenesis, we have generated mice overexpressing this mitochondrial T3 receptor under control of the 2.2-kb human α-skeletal actin promoter (HSA) flanked by chicken β-globin 5′HS4 insulator (Figure 1A). Insulators are used to reduce the number of transgenic founders required to obtain animals with an appropriate expression level [19]. Mouse oocytes were injected with the construct, and three mouse lines expressing the transgene were obtained. Northern-blot confirmed that p43 is overexpressed in each mouse line. Moreover, no p43 messenger is detected in the control line because p43 is synthesized by the use of internal initiation site of translation occuring in the TRα1 transcript (Figure 1B). Western-blot analysis revealed that Line 86 and 90 expressed approximately 8-fold more p43 than wild-type controls in quadriceps muscle mitochondria (Figure 1C). Expression of p43 in line 163 was increased about 2 fold. Most experiments were conducted using line 86. Expression of the transgene in various tissues was assessed by Northern-blot (Figure 1D). As expected, and in agreement with the previous characterization of the promoter HSA [17], [18], the p43 transgene was selectively expressed in skeletal muscle (Figure 1D). Moreover, in order to exclude the possibility that transgenic p43 could be also targeted into the nucleus and act on nuclear transcription, we isolated skeletal muscle nuclei from transgenic mice. We found higher amounts of p43 in whole muscle homogenate and in the crude nuclear fraction, but it was absent in the nuclear fraction (Figure 1E). In addition, the nuclear receptor TRα1 was detected in the crude nuclear fraction and in the nucleus (Figure 1E). These data indicate that in transgenic mice, p43 was targeted to the mitochondria but not into the nucleus.

### p43 enhances mitochondrial activity and mitochondriogenesis

**Figure 2:**
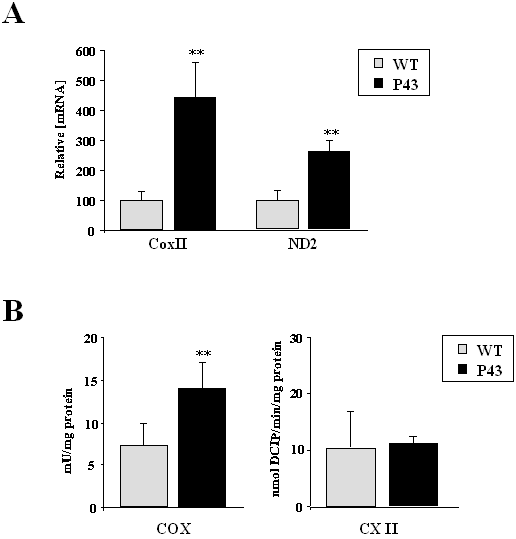
p43 overexpression increases mitochondrial transcription and mitochondrial activity. (A) Relative mRNA expression levels of mitochondrial transcripts (COXII and ND2) in quadriceps muscle from transgenic mice of the 86 line compared to wild-type animals at 2 months of age (n = 8 each group). **p<0.01. (B) Enzymatic activities of mitochondrial respiratory chain complexes in quadriceps muscle from transgenic mice of the 86 line compared to wild-type animals at 2 months of age (n = 8 each group). COX: cytochrome c oxydase; CXII: complex II. **p<0.01.

To validate *in vivo* the *in vitro* demonstration that p43 is a mitochondrial transcription factor, we studied by quantitative PCR, the expression of mRNA encoded by the mitochondrial genome. In agreement with our previous studies [11], we found that p43 overexpression in quadriceps muscle (oxido-glycolytic muscle) increased mitochondrial transcript expression as shown for COX II (+442%; p<0.01) and ND2 (+279%; p<0.01) (Figure 2A). We also measured the enzymatic activities of respiratory chain complexes on quadriceps muscle extracts. We observed that p43 overexpression induced a strong stimulation of cytochrome oxidase activity (COX) (+95%; p<0.01). However, complex II activity, including only nuclear encoded subunits, was not modified (Figure 2B).

**Figure 3:**
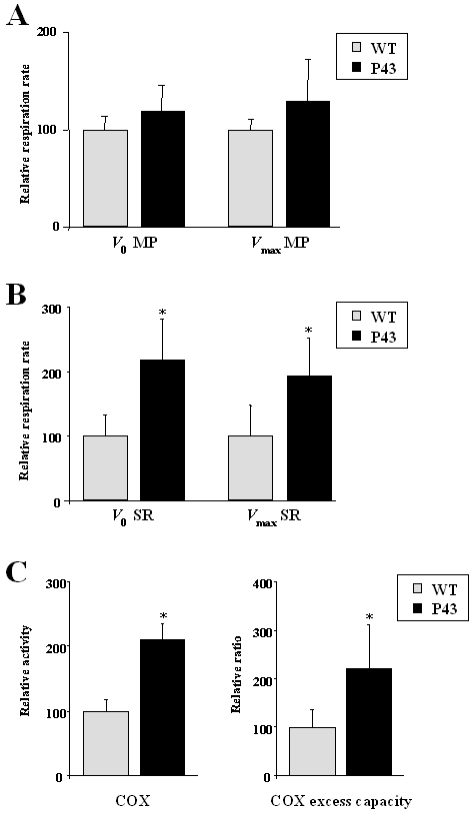
p43 overexpression increases mitochondrial respiration. (A–B) Relative respiration rate in permeabilized gastrocnemius muscle fibers from transgenic mice of the 86 line compared to wild-type animals at 2 months of age (n = 5 each group). Respiration parameters were recorded at 25°C using a Oroboros oxygraph. Resting respiration (*V*_0_) was initiated in the presence of complex I (5 mM malate and 5 mM pyruvate) or complex II substrates (10 mM succinate and 2.5 µM rotenone), and maximal ADP-stimulated respiration was measured with one addition of saturating ADP concentration (1 mM)(*V*_max_). MP: malate and pyruvate; SR: succinate and rotenone. *p<0.05. (C) Relative COX activity and excess COX capacity in permeabilized gastrocnemius muscle fibers from transgenic mice of the 86 line compared to wild-type animals at 2 months of age (n = 5 each group). COX activity was measured after addition of ascorbate and TMPD. COX: cytochrome c oxydase; Excess COX capacity: [*V*_max_(COX)−*V*_max_(malate+pyruvate)]/*V*_max_(malate+pyruvate). *p<0.05.

To assess the influence of p43 on skeletal muscle mitochondria oxygen consumption, we performed *in situ* measurements on isolated permeabilized fibers from gastrocnemius muscle (oxido-glycolytic muscle) collected on p43 transgenic mice and wild-type controls. Resting respiration (*V*_0_) was measured in the presence of substrates of complex I (malate/pyruvate) or II (succinate/rotenone); maximal ADP-stimulated respiration (*V*_max_) was measured under addition of saturating ADP concentration. Whereas no significant difference were recorded in the presence of complex I substrates (Figure 3A), we observed an up to two-fold increase in *V*_0_ (+118%; p<0.05) and *V*_max_ (+92%; p<0.05) respiration rate in the presence of complex II substrates in transgenic mice relatively to controls (Figure 3B). As previously shown, COX activity (+110%; p<0.05) was strongly increased in gastrocnemius isolated permeabilized fibers collected from transgenic mice (Figure 3C). Moreover, the excess COX capacity defined as [*V*_max_(COX)−*V*_max_(malate+pyruvate)]/*V*_max_(malate+pyruvate) [20] was also higher in p43 overexpressing animals than in controls (+120%; p<0.05) (Figure 3C). These data suggest that in gastrocnemius muscle, p43 overexpression favours the use of oxidative substrates.

**Figure 4:**
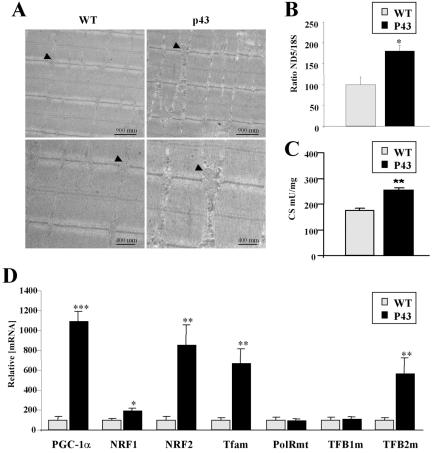
p43 stimulates mitochondrial biogenesis. (A) electronic microscopy experiments from longitudinal section taken from quadriceps muscle from transgenic mice of the 86 line compared to wild-type animals at 2 months of age (magnification ×10.000 and ×20.000). Arrows indicate mitochondria. (B) Relative mtDNA content in quadriceps muscle from transgenic mice of the 86 line compared to wild-type animals at 2 months of age (n = 8 each group). After extraction of muscle DNA, quantitative PCR reactions were performed using ND5 for mtDNA copy estimation, and 18S for the nuclear genome. Ratio ND5/18S was expressed as percent of control value. *p<0.05. (C) Citrate Synthase activity in quadriceps muscle from transgenic mice of the 86 line compared to wild-type animals at 2 months of age (n = 8 each group). CS: citrate synthase. **p<0.01. (D) Relative mRNA expression levels of the indicated genes from gastrocnemius muscle from transgenic mice of the 86 line compared to wild-type animals at 2 months of age (n = 8 each group). *p<0.05; **p<0.01; ***p<0.001.

To test the influence of p43 on mitochondrial biogenesis, we performed electron microscopy observations and assessment of mitochondrial DNA (mt-DNA) content relatively to nuclear DNA (ND5 and 18S) in quadriceps muscle. In electron microscopy studies, we found that p43 overexpression increases mitochondrial mass (Figure 4A). In addition, the ratio ND5/18S in quadriceps was significantly higher in trangenic mice than in wild type mice (+81%; p<0.05) (Figure 4B). In line with this observation citrate synthase activity commonly used as marker of mitochondrial biogenesis was increased in transgenic mice (+46%; p<0.01) (Figure 4C). These data convincingly suggest that p43 induces a marked stimulation of mitochondrial biogenesis. To better understand this influence, we have investigated the expression of genes regulating mitochondrial biogenesis. In quadriceps, we found a strong increase in the transcript levels encoding PGC-1α (+990%; p<0.001), NRF2 (+755%; p<0.01), Tfam (+566%; p<0.01), TFB2m (+468%; p<0.01) and more moderatly NRF1 (+91%; p<0.05) (Figure 4D). These data led us to conclude that p43 overexpression results in a stimulation of the expression of nuclear genes involved in mitochondrial biogenesis.

### p43 increases body temperature and decreases T3 plasma levels

**Figure 5:**
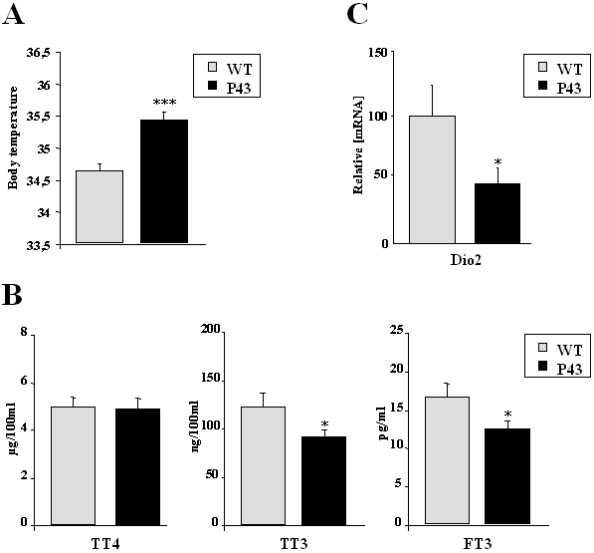
p43 overexpression increases body temperature. (A) Body temperature from transgenic mice of the 86 line compared to wild-type animals at 2 months of age. ***p<0.001. (B) Levels of total T4, total T3 and free T3 in serum from transgenic mice of the 86 line compared to wild-type animals at 2 months of age (n = 8 each group). T4, total T3 and free T3 were measured using a direct competitive radioimmunoassay from MP-Biomedicals. *p<0.05. (C) Relative mRNA expression levels of type 2 iodothyronine deiodinase (Dio2) in quadriceps from transgenic mice of the 86 line compared to wild-type animals at 2 months of age (n = 8 each group). *p<0.05.

It has been previously shown that TRα gene invalidation in mice induced a significant decrease in body temperature [21]. Interestingly, in 2 months old mice, we recorded an 0.8°C increase in body temperature in p43 overexpressing mice (p<0.001) (Figure 5A). This result clearly suggests that the influence of the TRα gene on thermogenesis is essentially mediated through p43 expression and is induced by mitochondrial activity.

More surprising was the observation indicating that p43 overexpression in muscle led to a significant decrease of total and free T3 levels in plasma (respectively −26% and −25%; p<0.05) (Figure 5B) without any changes in plasma T4 levels. In agreement with the possibility that T4 deiodination could be altered in transgenic mice, we found that Dio2 expression, a deiodinase selectively producing T3 from T4, was severly decreased by p43 overexpression in gastrocnemius muscle (−53%; p<0.05) (Figure 5C).

### p43 induces a shift toward the contractile slow type phenotype

**Figure 6:**
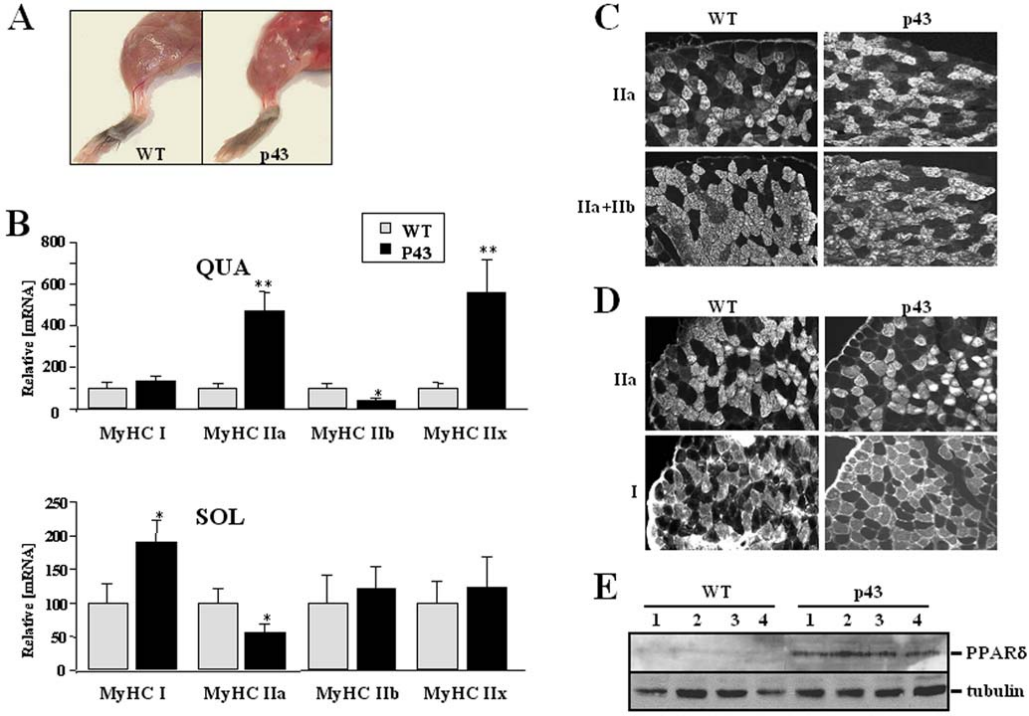
p43 increase muscle oxidative fibers. (A) Hindlimb from transgenic mice of the 86 line compared to wild-type animals at 2 months of age. (B) Relative mRNA expression levels of the four adult MyHC isoforms in the indicated muscle from transgenic mice of the 86 line compared to wild-type animals at 2 months of age (n = 8 each group). QUA: quadriceps; SOL: soleus. *p<0.05; **p<0.01. (C) Immunohistochemical analysis of gastrocnemius muscle from transgenic mice of the 86 line compared to wild-type animals at 2 months of age, using antibodies raised against MyHC Type IIa or IIa+IIb. (D) Immunohistochemical analysis of soleus muscle from transgenic mice of the 86 line compared to wild-type animals at 2 months of age, using antibodies raised against MyHC Type I or IIa. (E) PPARδ protein levels in quadriceps muscle mitochondria from transgenic mice of the 86 line versus wild-type animals, visualized by western-blot using an antibody raised against PPARδ. 50 µg of total protein extracts were analyzed.

In 2 month old transgenic mice, muscles were redder than in control animals (Figure 6A). This feature was recorded in all three mouse lines, despite differences in p43 expression levels. To determine whether this change in muscle color was associated with a fiber type switch, we measured the expression of the four adult MHCs transcripts. RNAs were isolated from quadriceps and soleus skeletal muscles from WT and transgenic mice, and assessed by quantitative PCR. In quadriceps muscle, MyHCIIb transcript levels were lower in transgenic mice than in control mice (−58%; p<0.05) (Figure 6B). In contrast, p43 overexpressing mice displayed a higher expression of MyHCIIx (+557; p<0.01) and MyHCIIa (+467%; p<0.01) than control animals (Figure 6B). No difference was observed for MyHCI. These data indicated that in quadriceps muscle, p43 overexpression induced a shift toward a slower contractile phenotype, with an increase in the expression of MyHC IIa and IIx, and a reduction of MyHCIIb. These findings were consistent with the increase in mitochondrial activity previously described. In soleus muscle, a muscle rich in type I and type IIa fibers, we found in transgenic mice an increase in the expression of MyHCI (+191%; p<0.05) associated with a reduction of type IIa (−44%; p<0.05) (Figure 6B). However, no changes were detected for MyHC IIx and IIb which are normally weakly expressed in soleus muscle. In agreement with these findings, on immuno-histological analysis of gastrocnemius muscle, we showed no difference in staining with an antibody raised against MyHC IIa and IIb (Figure 6C), whereas staining with an antibody only raised against MyHC IIa clearly indicated that IIa fibers were more abondant in transgenic than in control mice (Figure 6C). In addition, because almost no type I fibers were detected, these data indicate that type IIb fibers were predominantly replaced by type IIa and IIx fibers in gastrocnemius muscle. In transgenic soleus muscle, we found an increase in the number of type I fibers associated with a reduction of type IIa number (Figure 6D). It has been previously shown that PPARδ induces a shift toward slow oxidative fibers [22] as observed for p43 in this study. Interestingly, western-blot analysis revealed that PPARδ is strongly expressed in quadriceps muscle extracts from transgenic mice thus suggesting that PPARδ could be downstream targets of mitochondrial activity. These data are in agreement with our previous data showing that overexpression of p43 in the myoblast C2C12 cell line induced preferentially slow myosin expression [16]. Thus, relative to MyHC gene expression and mitochondrial activity, skeletal muscle of p43 overexpressing mice displays more oxidative and slow muscle fibers: in soleus muscle, MyHC IIa were partly replaced by type I fibers, and in gastrocnemius muscle, we found an increase in MyHC IIa and IIx fibers associated with a reduction of type IIb glycolytic fibers.

## Discussion

We have previously shown that p43 overexpression in myoblast stimulates terminal differentiation [14], [15]. The influence of p43 on mitochondrial activity and biogenesis [9], [11], and the fact that mitochondrial metabolism is deeply involved in the metabolic muscle-fiber phenotype led us to examine the potential role of p43 in the control of muscle fiber characreristics. To directly assess *in vivo* the role of p43, we generated transgenic mice expressing p43 under the control of the HSA muscle specific promoter [17], [18]. As expected, p43 is specifically overexpressed in muscle mitochondria from transgenic lines. P43 expression induced a rise in mitochondrial transcript levels as attested by measurement of COXII and ND2 mRNAs. In addition to this effect, p43 also increased respiratory chain activity as reflected by cytochrome c oxidase activity (COX). We have previously shown that p43 overexpression in CV1 cells or in fibroblasts increase mitochondrial biogenesis [9], [23]. As shown in electron microscopy observations, p43 overexpression increases mitochondrial mass. Moreover, the ratio mitochondrial DNA/nuclear DNA was substantially higher in trangenic mice than in wild type mice. These data indicate that p43 induces a marked stimulation of mitochondrial biogenesis. Therefore, these data are consistent with a role of p43 as an *in vivo* mitochondrial transcription factor involved in mitochondrial activity and biogenesis, as previously shown *in vitro* [9], [11]. In addition, the observation that p43 overexpression results in an increased expression of numerous genes involved in mitochondrial metabolism such as PGC-1α, NRF1, NRF2, Tfam, TFB2m, satisfactory explaines the stimulation of mitochondrial activity and biogenesis.

Study of *in situ* oxygen consumption performed on isolated permeabilized fibers from gastrocnemius muscle, clearly indicates that p43 increases the *V*_0_ and *V*_max_ respiration rate in the presence of complex II substrates, which are preferentially used in oxidative muscle. The increase in COX excess capacity in muscle of transgenic mice could indicate a stronger respiratory chain capacity. Previous results demonstrated a tight *in vivo* control of respiration by COX in skeletal muscle [24], indicating that p43 overexpression favours the use of oxidative substrates and a muscle fiber type switch. Assessement of the composition of muscle fibers confirms that p43 overexpression induces significant changes in the contractile phenotype of muscle fibers: in soleus muscle, MyHC IIa were partly replaced by type I fibers whereas in quadriceps muscle, we found an increase of MyHC IIa and IIx associated with a reduction in the number of type IIb glycolytic fibers. These data are in agreement with our previous data establishing that overexpression of p43 in the myoblast C2C12 cell line induced preferentially slow myosin [16]. The observation that the number of MyHC IIa and IIx fibers were up-regulated in fast muscles (quadriceps) and the number of MyHC I fiber was increased in slow muscles (soleus) of transgenic mice is in agreement with the hypothesis of a limited adaptative range theory where muscles from a slow developmental lineage have the capacity to adapt in the range of I↔IIa but not type IIb fibers, whereas muscles from a fast developmental lineage can adapt in the range of IIb↔IIx↔IIa but not type I fibers [25].

Our data reveal that p43, a mitochondrial T3 receptor can influence the acquisition of muscle fibers phenotype. We establish that p43 expression induces profound and coordinated increases in mitochondrial activity, mitochondrial biogenesis, leading to a shift toward slow oxidative fibers. Muscle phenotypes described in this study are remarkably similar to that reported in transgenic mice overexpressing either PGC-1α, calcineurin or PPARδ [22], [26], [27], [28]. However, in contrast to these proteins, p43 is a mitochondrial protein which directly acts on organelle activity, indicating that mitochondria by themselves are able to modify contractile and metabolic features of muscle fibers, and could act upstream of PGC-1α or PPARδ because both of these factors were up regulated in p43 transgenic mice.

This observation underlines that the mitochondrial-nucleus crosstalk could be involved in muscle specification. Several data obtained in xenopus oocytes or in human dermal fibroblasts clearly indicate that p43 overexpression modulates Ca^2+^ signalling [29] or increases mitochondrial reactive oxygen species production [23], two well known signaling molecules. It is probably through this crosstalk that p43 could increase the expression of numerous genes involved in mitochondrial biogenesis such as PGC-1α, NRF1, NRF2, Tfam, TFB2m, thus satisfactory explaining the changes of contractile and metabolic features of muscle fibers.

In addition, our results provides evidence that p43 overexpressing mice leads to an increase in body temperature (about 0.8°C). This result, associated with a previous study indicating that TRα gene invalidation in mice induced a significant decrease in body temperature [21], clearly suggests that the influence of the TRα gene on thermogenesis is essentially mediated through p43 expression and is induced at the level of mitochondrial activity. Moreover, the observation that p43 overexpression in muscle led to a significant decrease T3 levels in plasma indicates that hormone and p43 have opposite effects in body temperature.

**Figure 7:**
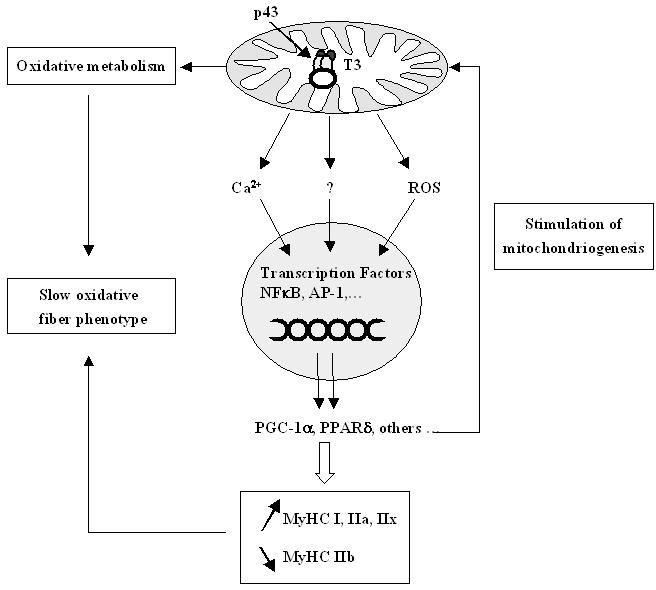
A positive regulatory loop between mitochondrial activity and nuclear genes such as PGC-1α and PPARδ could be able to coordinate the increase in oxidative metabolism and the expression of slower myosin isoforms. A stimulation of mitochondrial activity induced by p43, could modulate Ca^2+^ signalling and mitochondrial reactive oxygen species production, which are well known for altering the activity of some transcriptional factors such as NFκB and AP-1. In this way mitochondrial activity could affect nuclear gene expression of proteins such as PGC-1α and PPARδ which are involved in muscle specification and in the regulation of mitochondrial biogenesis. This positive regulatory loop could be deeply involved in the determination of muscle metabolic and contractile phenotypes.

In conclusion, these results establish that a stimulation of mitochondrial activity induced by p43 overexpression induces a shift toward a slower/oxidative phenotype in muscle fibers. It has been reported that PGC-1α and PPARδ overexpression have probably to be considered as major regulators of muscle phenotype. Interestingly, our own data indicate that these factors could be downstream targets of mitochondrial activity. However, as they are also involved in the regulation of mitochondriogenesis, a positive regulatory loop occuring between mitochondrial activity, PGC-1α and PPARδ could be deeply involved in the determination of muscle metabolic and contractile phenotypes (Figure 7). In addition, it appears that the direct T3 mitochondrial pathway mediated by p43, by inducing or stimulating this regulatory loop, is probably able to coordinate the increase in oxidative metabolism and the expression of slower myosin isoforms. Finally, these results also provide evidence that the thermogenic T3 influence is probably essentially mediated by the mitochondrial T3 receptor.

## Materials and Methods

### Transgene construct

The mouse p43 coding sequence was amplified using the following primers: 5′mp43, CCCTGAAAAGCAGCATGTCA; 3′mcea1250, AAGATCTGCCGCCTGAGGCTTTAGA, and cloned in a pGEM-T vector. The 1.0 kb EcoRI fragment from pGEM-mp43 was inserted in the pGS-HSA plasmid (kindly provided by J. Melki)[18] containing the 2.2 kb fragment of the human α-skeletal actin promoter (HSA)[17], [30], a beta globin intron and SV40 polyadenylation site. The NotI fragment of the pGS-HSA-mp43 plasmid was inserted in pBS3isol plasmid containing three copies the 5′HS4 fragment from the chicken β-Globin gene locus (kindly provided by LM. Houdebine)[31].

### Generation of founders and screening of transgenic mice

All animals experiments were performed according to European directives (86/609/CEE). The transgene was excised from pBSisol/HSA-mp43/2isol on a KpnI fragment. DNA was micro-injected into fertilized oocytes of F1 mice (C57BL/6JxCBA). Screening of founder mice and their offspring for stable transmission through the germline was done by PCR and Southern-Blotting. DNA was extracted from tail biopsy in lysis buffer (100 mM Tris-HCL pH 7.5, 1 mM EDTA, 0.2% SDS, 200 mM NaCl, 100 µg proteinase K) overnight at 56°C followed by phenol extraction, isopropanol precipitation and resuspension in H_2_O. A 531 bp fragment corresponding to the endogenous c-erbAα locus was amplified using the following primers: 5′Exon6, AGGAGGAGATGATTCGCTCACT; 3′intron 6, CTTGGTGTTGGGTAACTTAGTGCA. A 283 bp fragment corresponding to the transgene was amplified using the following primers: 5′Exon6, AGGAGGAGATGATTCGCTCACT; 3′ceaE7, CTCGGAGAACATGGGCAGTTTT. The PCR was performed in Promega reaction buffer containing 400 ng of genomic DNA, 1.5 mM MgCl_2_, 0.3 µM of each primer, 0.3 mM of each dNTP and 1.25 u of TAQ polymerase in a final volume of 25 µl. Thirty cycles were performed with an annealing temperature of 56°C. For Southern-Blot analysis, 10 µg of total genomic DNA was digested using EcoRI restriction enzyme and transferred to a Nytran supercharge membrane (Schleicher & Schuell) following standard procedures.

### Isolation of skeletal muscle nuclei

Muscles are dissected, weighed and minced. Then muscles are homogenized in a 0.25 M STEAKM buffer (0.25 M sucrose, 25 mM KCl, 50 mM triethanoloamine–HCl pH 7.5, 5 mM MgCl_2_) (2 ml of sucrose solution per gm of muscle) with a Potter-Elvehjem homogenizer. The muscle homogenate is filtered through gaze and diluted 5 times with 0.25 M Sucrose solution (fraction 1, whole muscle homogenate-I). The homogenate is then filtered through a cell strainer (pore size, 40 µm) (fraction 2, whole muscle homogenate-II). The muscle homogenate-II is centrifuged at 800g for 15 minutes at 4°C. The pellet was homogenized in 0.25 M STEAKM buffer (Fraction 3, crude nuclear fraction), supplemented with two volumes of 2.3 M STEAKM buffer, and layered on top of 10 ml of 2.5 M STEAKM and centrifuged for 1 h at 124 000*g*. The band at the interface is enriched in plama membrane and mitochondria (Fraction 4). The pellet contains the nuclei.

### Histological analysis

Fresh muscles were immersed in a solution of 3.5% glutaraldehyde in phosphate buffer (0.1 M, pH 7.4) overnight at 4°C. They were then rinced in phosphate buffer and post-fixed (1% osmic acid, 0.8% potassium ferrocianide) for 2 h in the dark and at room temperature. After two rinces in a phosphate buffer, muscles were dehydrated in a graded series of ethanol solutions (30–100%). The cells were embedded in EmBed 812 DER 736. Thin sections (85 nm; Leica-Reichert Ultracut E) were collected at different levels of each block. These sections were counterstained with uranyl acetate and lead citrate and observed using a Hitachi 7100 transmission electron microscope in the Centre de Ressources en Imagerie Cellulaire de Montpellier (France).

For all other histologies, tissues were collected, embedded with OCT matrix, and immediately frozen in isopentane cooled in liquid nitrogen. Ten µm thick serial sections were obtained and processed for immunohistochemichal staining with monoclonal antibodies raised against MyHC type I; type IIa and Type IIa+IIb (Alexis Biochemical). Briefly, the muscle sections were incubated with the antibody for 1 hour at 37°C. After washing with phosphate-buffered saline, the second antibody, rabbit anti-mouse IgG labelled with cyanine (Fluoprobes) diluted 1∶50 v/v in phosphate-buffered saline, was applied for 30 min at 37°C. After further washing, the sections were fixed with mowiol. Immunohistochemical controls were not incubated with anti-MyHC antibodies.

### Gene expression studies

Total RNA were isolated from mouse tissues using the Trizol method (invitrogen). Samples were reverse transcribed using superScript first-Strand synthesis System (invitrogen), and quantitative PCR reactions were performed on the cDNAs in the presence of fluorescent dye (SYBR Green, Bio-Rad). The following primers were used: COXII (forward, TCTCCCCTCTCTACGCATTCTA; reverse, ACGGATTGGAAGTTCTATTGGC); ND5 (forward, GGCAGACGAACAAGACATCCGAAA; reverse, GCTAGGCGTTTGATTGGGTT); POLRMT (forward, CTCCTCCCACATGATGCTGAC; reverse, AATTGCTCGCGGCATACCT); TFAM (forward, AATGTGGAGCGTGCTAAAAGC; reverse, GCTGAACGAGGTCTTTTTGGT); TFB1 (forward, ACCGAGGGCTTGGAATGTTA; reverse, TGGATCAATGTCTGCCAACTGT ); TFB2 (forward, TTTGGCAAGTGGCCTGTGA; reverse, CCCCGTGCTTTGACTTTTCTA); NRF-1 (forward, CCACGTTGGATGAGTACACG; reverse, CTGAGCCTGGGTCATTTTGT); NRF-2 (forward, CCGCTACACCGACTACGATT; reverse, ACCTTCATCACCAACCCAAG), PGC-1α (forward, GGAGCCGTGACCACTGACA; reverse, TGGTTTGCTGCATGGTTCTG); MyHC-I (forward, CCTTGGCACCAATGTCCCGGCTC; reverse, GAAGCGCAATGCAGAGTCGGTG); MyHC-IIa (forward, ATGAGCTCCGACGCCGAG; reverse, TCTGTTAGCATGAACTGGTAGGCG); MyHC-IIx (forward, AAGGAGCAGGACACCAGCGCCCA; reverse, ATCTCTTTGGTCACTTTCCTGCT); MyHC-IIb (forward, GTGATTTCTCCTGTCACCTCTC; reverse, GGAGGACCGCAAGAACGTGCTGA); DIO2 (forward, TCCCTCACCCCCCTCCCAACC; reverse, GCCCCATCAGCGGTCTTCTCC); 18S RNA (forward, GGACCAGAGCGAAAGCATTT; reverse, GGAGGACCGCAAGAACGTGCTGA). DNA product of the expected size was confirmed for each primer pair. After normalization by 18S, all results are expressed as percent of control as means±SEM. Student's t-test was used to determine all p values.

### Measurement of mtDNA copy number

The mtDNA content is the mtDNA copy number normalized to the copy number of a nuclear gene. After extraction of muscle DNA, quantitative PCR reactions were performed using ND5 (forward, GGCAGACGAACAAGACATCCGAAA; reverse, GCTAGGCGTTTGATTGGGTT) for mtDNA copy estimation, and 18S for the nuclear genome. All results are expressed as percent of control as means±SEM. Student's t-test was used to determine all p values.

### Protein studies

50 µg of mitochondrial extracts or total protein extacts were electrophoresed onto 10% SDS-Page gels and blotted onto PDVF membranes. The presence of p43 and PPARδ was assessed using respectively RHTII and anti-PPARδ antisera as previously described [9], [12]. The presence of α-tubulin used for normalization was assessed using a monoclonal anti-α-tubulin (SIGMA). The presence of proteins were revealed using a chemioluminescent Western blot procedure (ECF kit, Amersham) and analyzed with a PhosphorImager (Molecular dynamics).

### Enzymatic activities of mitochondrial complexes

Muscle enzyme activites were measured from whole quadriceps homogenates. Proteins concentration was measured using the Bio-Rad protein assays kit. Citrate synthase activity was measured as described [32]. Cytochrome oxidase was measured as described [33] and was expressed in mU/mg protein. Student's t-test was used to determine all p values.

### Mitochondrial respiration on isolated fibers

Saponin-permeabilized muscle fibers were prepared from gastrocnemius muscle as previously described [34]. Briefly, thin fibre bundles were excised along the fiber orientation to avoid mechanical damage to the cells. Fibers were carefully separated from each other using sharp-ended forceps and needles in cooled solution A (containing in mm: CaK_2_EGTA 2.77, K_2_EGTA 7.23, MgCl_2_ 6.56, DTT 0.5, potassium 2-(*N*-morpholino) ethansulphonate (K-Mes) 50, imidazole 20, taurine 20, ATP 5.3, phosphocreatine 15, pH 7.1 adjusted at 4°C, free Ca^2+^ concentration 0.1 µM, a condition which prevents contraction of the bundles) until bundles of roughly 20–30 fibers were obtained with only small areas of contact between them. Fibers were then permeabilized by incubation in solution A supplemented with 50 µg ml^−1^ saponin with gentle shaking for 30 min at 4°C. To completely remove all metabolites, including trace amounts of ADP, fibres were washed three times in respiration solution B (containing in mM: CaK_2_EGTA 2.77, K_2_EGTA 7.23, MgCl_2_ 1.38, DTT 0.5, K-Mes 100, imidazole 20, taurine 20, and K_2_HPO_4_ 3, pH 7.1 adjusted at 25°C, free Ca^2+^ concentration 0.1 µM) supplemented with bovine serum albumin (2 mg ml^−1^). Respiratory parameters of fiber bundles (20–30 fibers) were recorded at 25°C using a Oroboros oxygraph. Resting respiration (*V*_0_) was initiated in the presence of complex I (5 mM malate and 5 mM pyruvate) or complex II substrates (10 mM succinate and 2.5 µM rotenone), and maximal ADP-stimulated respiration was measured with one addition of saturating ADP concentration (1 mM)(*V*_max_). The excess COX capacity is defined as [*V*_max_(COX)−*V*_max_(malate+pyruvate)]/*V*_max_(malate+pyruvate) [20]. Specific respiration rate linked to COX was determined, following complex III inhibition, as respiration rate with 5 mM ascorbate and 1 mM TMPD, corrected for residual respiration after cyanide addition (1 mM). Similarly, complex I-linked respiration was determined as *V*_max_ using malate and pyruvate as substrates, corrected for residual respiration after rotenone (10 µM) addition. All results are expressed as percent of control as means±SEM. Student's t-test was used to determine all p values.

### Statistical analyses

All results are presented as means±SEM, or as percentages. The significance of the difference between groups was evaluated with Student's t-test. *p<0.05; **p<0.01; ***p<0.001. p<0.05 was considered significant.
